# NF-κB Signaling Regulates Expression of Epstein-Barr Virus BART MicroRNAs and Long Noncoding RNAs in Nasopharyngeal Carcinoma

**DOI:** 10.1128/JVI.00613-16

**Published:** 2016-06-24

**Authors:** Rob J. A. Verhoeven, Shuang Tong, Gaohong Zhang, Jingfeng Zong, Yixin Chen, Dong-Yan Jin, Mei-Ru Chen, Jianji Pan, Honglin Chen

**Affiliations:** a State Key Laboratory for Emerging Infectious Diseases, Department of Microbiology and the Collaborative Innovation Center for Diagnosis and Treatment of Infectious Diseases, The University of Hong Kong, Hong Kong SAR, People's Republic of China; b Department of Radiation Oncology, Fujian Provincial Cancer Hospital, Provincial Clinical College of Fujian Medical University and Fujian Provincial Key Laboratory of Translational Cancer Medicine, Fuzhou, Fujian, People's Republic of China; c National Institute of Diagnostics and Vaccine Development in Infectious Diseases, School of Life Sciences, Xiamen University, Xiamen, Fujian, People's Republic of China; d School of Biomedical Science, The University of Hong Kong, Hong Kong SAR, People's Republic of China; e Graduate Institute and Department of Microbiology, College of Medicine, National Taiwan University, Taiwan; University of Southern California

## Abstract

Epstein-Barr virus (EBV) expresses few viral proteins in nasopharyngeal carcinoma (NPC) but high levels of BamHI-A rightward transcripts (BARTs), which include long noncoding RNAs (lncRNAs) and BART microRNAs (miRNAs). It is hypothesized that the mechanism for regulation of BARTs may relate to EBV pathogenesis in NPC. We showed that nuclear factor-κB (NF-κB) activates the BART promoters and modulates the expression of BARTs in EBV-infected NPC cells but that introduction of mutations into the putative NF-κB binding sites abolished activation of BART promoters by NF-κB. Binding of p50 subunits to NF-κB sites in the BART promoters was confirmed in electrophoretic mobility shift assays (EMSA) and further demonstrated *in vivo* using chromatin immunoprecipitation (ChIP) analysis. Expression of BART miRNAs and lncRNAs correlated with NF-κB activity in EBV-infected epithelial cells, while treatment of EBV-harboring NPC C666-1 cells with aspirin (acetylsalicylic acid [ASA]) and the IκB kinase inhibitor PS-1145 inhibited NF-κB activity, resulting in downregulation of BART expression. Expression of EBV LMP1 activates BART promoters, whereas an LMP1 mutant which cannot induce NF-κB activation does not activate BART promoters, further supporting the idea that expression of BARTs is regulated by NF-κB signaling. Expression of LMP1 is tightly regulated in NPC cells, and this study confirmed that miR-BART5-5p downregulates LMP1 expression, suggesting a feedback loop between BART miRNA and LMP1-mediated NF-κB activation in the NPC setting. These findings provide new insights into the mechanism underlying the deregulation of BARTs in NPC and identify a regulatory loop through which BARTs support EBV latency in NPC.

**IMPORTANCE** Nasopharyngeal carcinoma (NPC) cells are ubiquitously infected with Epstein-Barr virus (EBV). Notably, EBV expresses very few viral proteins in NPC cells, presumably to avoid triggering an immune response, but high levels of EBV BART miRNAs and lncRNAs which exhibit complex functions associated with EBV pathogenesis. The mechanism for regulation of BARTs is critical for understanding NPC oncogenesis. This study provides multiple lines of evidence to show that expression of BARTs is subject to regulation by NF-κB signaling. EBV LMP1 is a potent activator of NF-κB signaling, and we demonstrate that LMP1 can upregulate expression of BARTs through NF-κB signaling and that BART miRNAs are also able to downregulate LMP1 expression. It appears that aberrant NF-κB signaling and expression of BARTs form an autoregulatory loop for maintaining EBV latency in NPC cells. Further exploration of how targeting NF-κB signaling interrupts EBV latency in NPC cells may reveal new options for NPC treatment.

## INTRODUCTION

Epstein-Barr virus (EBV) is a common human herpesvirus which infects more than 95% of adults worldwide (1). Besides causing lifelong persistent latent infections in humans, EBV is associated with several malignancies, including Burkitt's lymphoma (2), Hodgkin's disease (3), nasal NK/T cell lymphoma (4, 5), and epithelial tumors like nasopharyngeal carcinoma (NPC) and gastric carcinoma (GC) (6, 7). EBV is one of the few human viruses which can undergo true latency in which viral gene expression is stringently restricted, except for that of a few essential viral antigens, and which produces virtually no detectable viral particles *in vivo* (8). Based on the spectrum of viral gene expression, different latency forms, designated 0, I, II, and III, have been characterized in EBV infection and in EBV-associated tumors (9). While most EBV latent antigens are expressed only in the latency III form seen in EBV-associated posttransplant lymphoproliferative diseases, it is notable that in all forms of latency, EBV expresses small noncoding RNAs (EBERs) and a family of BamHI-A rightward transcripts (BARTs). EBERs have been widely used in pathological diagnosis of NPC and are suggested to interfere with host interferon expression through interaction with the PKR pathway (10, 11). The role of BARTs in EBV-infected cells has remained mostly unknown until recent years. BARTs were first identified as large multispliced transcripts in NPC tissues and were later found to be expressed in EBV-infected cells and EBV-associated tumors (12–16). In addition to the family of BART mRNAs which are produced by alternative splicing (17), there are two clusters of microRNAs (miRNAs) generated from introns of BARTs (18). However, attempts to identify protein expression from putative open reading frames in BART mRNAs have failed to generate any convincing findings (19). Nevertheless, BART miRNAs have been revealed to possess diverse functions, and BART mRNAs are likely to be functional long noncoding RNAs (lncRNAs) (20–23). Similar to EBNA1, which is essential for EBV latent replication, BARTs are consistently expressed at high levels in NPC (13, 14). BART expression is suggested to be deregulated in EBV-associated malignancies (24). We previously reported that BARTs are expressed from two promoters, P1 and P2, with the P1 promoter as the predominant initiation site (25, 26). Several potential binding sites for transcription factors such as CCAAT/enhancer binding protein (C/EBP), c-Myc, SP1, AP1, and interferon regulatory factor (IRF) have been identified around the P1 and P2 promoters. C/EBP and c-Myc were found to positively regulate P2 activity, and IRF-5 and -7 appear to regulate P1 activity (25). Another study showed that in EBV-infected B cell lines, methylation may play a role in regulating expression from BART promoters (27). It is not yet known how BART promoters are regulated to maintain the high-level expression of BARTs seen in NPC.

It is postulated that aberrant cellular signaling and viral functions may jointly contribute to regulate and maintain BART expression in EBV-associated tumors. In NPC cells, constitutive aberrant activation of STAT3 and nuclear factor-κB (NF-κB) has been described (28–30). The latent membrane protein 1 (LMP1) gene is a major EBV oncogene, and LMP1 functions as a constitutively active tumor necrosis factor (TNF) receptor, capable of activating several signaling pathways, including the NF-κB pathway (31). LMP1 signaling is important to the survival of latently infected cells because it upregulates antiapoptotic proteins and provides growth signals (32). LMP1 is also reported to upregulate its own expression through the NF-κB signaling pathway (33). It is intriguing that levels of LMP1 expression in NPC cells were found to be variable and even undetectable in some cases. Because LMP1 is able to activate several signaling pathways which strongly provoke the host immune response, it is possible that NPC cells adopt a mechanism to modulate the level of LMP1 expression in order to evade host immune surveillance. Interestingly, several BART miRNAs have been reported to downregulate LMP1; this may control LMP1 expression in EBV-infected tumor cells (34, 35).

We found putative NF-κB regulatory motifs located in the BART promoter region in addition to the previously characterized C/EBP and c-Myc regulatory elements. Since activated NF-κB is constitutively present in NPC cells, it seems possible that aberrantly activated NF-κB may play a role in upregulating expression of BARTs in these cells. To verify this hypothesis, we provide evidence showing that the BART promoters respond positively to NF-κB activation and that LMP1 can upregulate BART expression through the NF-κB pathway. In addition, BART expression can be downregulated by treatment with inhibitors which block NF-κB signaling. We further confirmed that BART miRNAs downregulate LMP1 expression through targeting of the 3′ untranslated region (UTR). A mechanism is proposed in which LMP1 and BART miRNA expression forms an autoregulatory loop through NF-κB signaling in NPC cells; this mechanism is likely to contribute to EBV latency and to the establishment and progress of NPC malignancy.

## MATERIALS AND METHODS

### Cell lines and cell culture conditions.

Human embryonic kidney (HEK) 293T cells, NPC CNE2, HeLa-Bx1, and HEK 293T virus-packaging Phoenix cells were maintained in Dulbecco's minimal essential medium (Gibco) supplemented with 10% fetal bovine serum (FBS) and 1% penicillin-streptomycin (P-S). The EBV-positive NPC cell line C666-1 was grown in RPMI 1640 medium (Gibco) supplemented with 10% FBS and 1% P-S, and the EBV-positive GC cell line AGS-Bx1 was cultured in F-12K nutrient mixture (Gibco) supplemented with 10% FBS and 1% P-S. C666-1 cells were treated for 48 h with 4 mM aspirin (acetylsalicylic acid [ASA]; Sigma) or 0.2 mM PS-1145 dihydrochloride (Santa Cruz) to inhibit NF-κB activity. All cells were cultured at 37°C with 5% CO_2_.

### Plasmids.

BART promoter reporter plasmids were constructed by cloning the inserts from the previously described BART P1 and P2 promoter plasmids (25) into the pGL2 (Amersham Pharmacia) luciferase reporter plasmid. The LMP1 expression vector was constructed by cloning the coding region of LMP1 into a V5-tagged pSG5 vector. The pcDNA3.1-EGFP (where EGFP is enhanced green fluorescent protein) expression vectors containing NF-κB p50 and p65 and the IκBα expression plasmid containing the IκBα-AA mutation and NF-κB-responsive reporter were kindly provided by Zhenguo Wu (Hong Kong University of Science and Technology, Hong Kong, People's Republic of China) (36). NF-κB p65 was also subcloned into a 5′ Flag-tagged pcDNA3.1 vector in this study. For the construction of cell lines stably expressing LMP1, we cloned the LMP1 gene including its 3′ untranslated region (3′ UTR) (GenBank accession number V01555.2; nucleotides 167120 to 169474) into the pBABE-Puro vector (Cell Biolabs, Inc.). Full-length and truncated forms of the 3′ UTR of LMP1 (GenBank accession number V01555.2; nucleotides 167120 to 168159) were cloned into the cloning site downstream of firefly luciferase in a pmirGLO Dual-Luciferase miRNA Target Expression Vector (Promega). Mutagenesis of the BART promoter and LMP1 was performed using a QuikChange site-directed mutagenesis kit (Stratagene), as described by the manufacturer. Primers are listed in Table 1.

**TABLE 1:** Oligonucleotides used in this study

Oligonucleotide	Sequence (5′–3′)^*a*^
pBABE-LMP1+3′UTR-BamHI-S	CGCGGATCCATGGAACACGACCTTGAGAG
pBABE-LMP1+3′UTR-EcoRI-AS	CCGGAATTCCTCCTCCCAACGCGTTTCTG
pmiRGLO-LMP1-XbaI-S	CTAGTCTAGACCTTTCTTTACTTCTAGGCA
pmiRGLO-LMP1-SalI-AS	ACGCGTCGACCTCCTCCCAACGCGTTTCTG
pmiRGLO-LMP1-168099-XbaI-S	CTAGTCTAGAGACTGACTCTCCCTCCAT
pmiRGLO-LMP1-168051-XbaI-S	CTAGTCTAGAACTGGCACACACTCCCTT
LMP1-Y384ID-S	GCCCAGTTCAGCTAAGCATC---GACTAAGGATCCAGATC
LMP1-Y384ID-AS	GATCTGGATCCTTAGTC---GATGCTTAGCTGAACTGGGC
ChIP-BART-S	CCTGCTTCTCAGGCCTAAAT
ChIP-BART-AS	CAGCTTGAAAAATGGCAACC
ChIP-IL8up-S	TCCCTAAGTCACTTTCTTCAAGTTGC
ChIP-IL8up-AS	CGTGCATTTAATTGTGTCTTGTGG
miR-control-top	5Phos/rCrUrArGrUrArUrGrArCrUrArGrUrArUrGrArUrCrCrGrG
miR-control-bottom	rGrGrArUrCrArUrArCrUrArGrUrCrArUrArCrUrArGrArC
miR-BART3-3p-top	5Phos/rCrGrCrArCrCrArCrUrArGrUrCrArCrCrArGrGrUrGrU
miR-BART3-3p-bottom	rArCrCrUrGrGrUrGrArCrUrArGrUrGrGrUrGrCrGrCrU
miR-BART5-5p-top	5Phos/rCrArArGrGrUrGrArArUrArUrArGrCrUrGrCrCrCrArUrGrC
miR-BART5-5p-bottom	rArUrGrGrGrCrArGrCrUrArUrArUrUrCrArCrCrUrUrGrArG
miR-BART7-3p-top	5Phos/rCrArUrCrArUrArGrUrCrCrArGrUrGrUrCrCrArGrGrG
miR-BART7-3p-bottom	rCrUrGrGrArCrArCrUrGrGrArCrUrArUrGrArUrGrCrA
miR-BART13-3p-top	5Phos/rUrGrUrArArCrUrUrGrCrCrArGrGrGrArCrGrGrCrUrGrA
miR-BART13-3p-bottom	rArGrCrCrGrUrCrCrCrUrGrGrCrArArGrUrUrArCrArGrA
miR-BART14-3p-top	5Phos/rUrArArArUrGrCrUrGrCrArGrUrArGrUrArGrGrGrArU
miR-BART14-3p-bottom	rCrCrCrUrArCrUrArCrUrGrCrArGrCrArUrUrUrArCrA
F-V(A/B) (BARF0/RPMS1A)	TCTTCTAGAGGACGCAGGATATCTGCAGGATCAGAGG
F-V (RPMS1/A73)	GAAAAGCTTGGGATTAATGCCTGGACCCTCACCAG
R-VII(D) (RPMS1/RPMS1A)	AGGGGATCCCCCGCCACCACGGTGCAGCCTAC
R-VII(A/B) (BARF0/A73)	AGGGGATCCGCACCCCCAGTACCGGGCCATCCG
F-GAPDH	GAAGGTGAAGGTCGGAGTA
R-GAPDH	GAAGATGGTGATGGGATTTC
F-EBV-BamH-W	CCCAACACTCCACCACACC
R-EBV-BamH-W	TCTTAGGAGCTGTCCGAGGG
EBV-BamH-W probe	(6 FAM)CACACACTACACACACCCACCCGTCTC(TAM)

a Underlining indicates altered nucleotides, hyphens indicate deleted nucleotides, 5Phos indicates a 5′ phosphate end, and r indicates RNA. 6 FAM is 6-carboxyfluorescein, and TAM is 6-carboxytetramethylrhodamine.

### EMSA.

Nuclear extracts for electrophoretic mobility shift assay (EMSA) were prepared from HEK 293T cells transfected with pcDNA3.1-EGFP-p50 and pcDNA3.1-Flag-p65. Cells were lysed using a sucrose buffer containing 0.5% NP-40, and the nuclei were washed and then resuspended in a hypotonic buffer. The nucleoplasm was extracted using a hypertonic buffer, and nuclear extracts were stored at −80°C following protein quantification. EMSA 5′ IRDye 700-labeled oligonucleotides were purchased from Integrated DNA Technologies and annealed to obtain double-stranded DNA probes. Binding reactions were performed as described by the manufacturer, and gels were visualized using an Odyssey infrared EMSA kit (Li-Cor Biosciences). Supershift experiments were performed with 2 μg of rabbit anti-p50 (sc-7178; Santa Cruz), rabbit anti-p65 (sc-7151; Santa Cruz), or rabbit control IgG (ab46540; Abcam).

### ChIP assay.

Briefly, HEK 293T cells were transfected with the BART promoter plasmid BART P1, pcDNA3.1-EGFP-p50, and pcDNA3.1-FLAG-p65 and were harvested after 48 h. The chromatin immunoprecipitation (ChIP) extract was sonicated into between 100- and 750-bp DNA fragments using a Sonicator S-4000 (Misonix). For immunoprecipitation, 5 μl of rabbit anti-GFP (A6455; Invitrogen), 5 μg of rabbit anti-Flag (F7425; Sigma) or 5 μg of rabbit control IgG (ab46540; Abcam) was used, and antibody-protein-DNA complexes were pulled down using Dynabeads protein A (Invitrogen). The level of immunoprecipitated DNA was determined by real-time PCR using a primer pair that amplified the BART P1 promoter region, including the putative NF-κB site, and a primer pair covering a genomic region about 5 kb upstream of the interleukin-8 (IL-8) promoter that served as a negative control (Table 1).

### Luciferase reporter assay.

HEK 293T cells were seeded at a density of approximately 70% in 24-well and 48-well plates a day before transfection and subsequently transfected using TransIT-LT1 transfection reagent (Mirus) or Lipofectamine 2000 (Invitrogen). For data normalization purposes, the plasmid phRL-TK (Promega) expressing Renilla luciferase was cotransfected with the firefly reporter plasmid in each experiment. For miRNA reporter assays, 25 ng of pmirGLO, with or without an insert, was cotransfected with 50 nM RNA duplexes.

### Immunoblotting.

Membranes were incubated overnight with primary antibodies at a dilution of 1:1,000. The antibodies used for immunoblotting include rabbit anti-NF-κB p50 (sc-7178; Santa Cruz), rabbit anti-NF-κB p65 (sc-7151; Santa Cruz), rabbit anti-Bcl-3 (sc-185; Santa Cruz), mouse anti-β-tubulin (sc-55529; Santa-Cruz), rabbit anti-IκBα (9242S; Cell Signaling), mouse anti-phospho-IκBα (5A5; Cell Signaling), mouse anti-lamin A+C (ab8984; Abcam), and mouse anti-LMP1 (OT21C; kindly provided by Jaap Middeldorp, VU University, Amsterdam, The Netherlands). Membranes were then incubated with IRDye 700-labeled donkey anti-mouse or anti-rabbit or IRDye 800-labeled donkey anti-mouse (Li-Cor Biosciences) at a 1:5,000 dilution. Blots were detected using an Odyssey infrared imaging system.

### Construction of stable cell lines expressing LMP1.

Virus-packaging HEK 293T Phoenix cells were transfected with a pBABE-LMP1-3′ UTR or pBABE-empty vector. Infected cells were selected by culturing in medium supplemented with 2.5 μg/ml puromycin.

### RT and real-time PCR amplification.

RNA was extracted from cells using RNAiso Plus reagent (TaKaRa) and reverse transcribed with gene-specific reverse transcription (RT) primers or random primers using a high-capacity cDNA reverse transcription kit (Applied Biosystems). The real-time PCR was performed using SYBR Premix Ex Taq (Tli RNase H Plus) mix (TaKaRa) in a LightCycler 480 instrument (Roche). Primers used to amplify the alternatively spliced BART RNAs, BARF0, RPMS1, RPMS1A, and A73, are listed in Table 1, and gene expression was normalized to that of glyceraldehyde-3-phosphate dehydrogenase (GAPDH). The real-time PCR amplification was performed with miRNA-specific TaqMan probes using SensiFAST Probe Lo-ROX mix (Bioline) in a LightCycler 480 instrument. Primers and probes used for BART miRNA detection were described previously (37). To determine EBV copy number, a 75-bp fragment of the BamH-W region (GenBank accession number V01555.2; nucleotides 17721 to 17796) was amplified by quantitative PCR (qPCR) from phenol-chloroform-extracted DNA. The same fragment was cloned into the TA cloning vector pMD18-T, which was then serially diluted to obtain a standard curve.

### Multiplex reverse transcription and real-time PCR amplification of BART miRNAs.

All of the miRNA sequences used in this study were obtained from the Sanger Institute database (miRBase). RNA was reverse transcribed in a mix containing two specific stem-loop primers using a TaqMan microRNA reverse transcription kit (Applied Biosystems). Real-time PCRs were performed with miRNA-specific TaqMan probes using SensiFAST Probe Lo-ROX mix in a LightCycler 480 instrument. Primers and probes used for BART miRNA detection have been described previously (37).

### Statistics.

The statistical analysis in this study was performed using GraphPad Prism, version 5, software. Significance values (*P* values) were calculated using a two-tailed Student's *t* test.

## RESULTS

### NF-κB positively regulates BART promoters.

**FIG 1:**
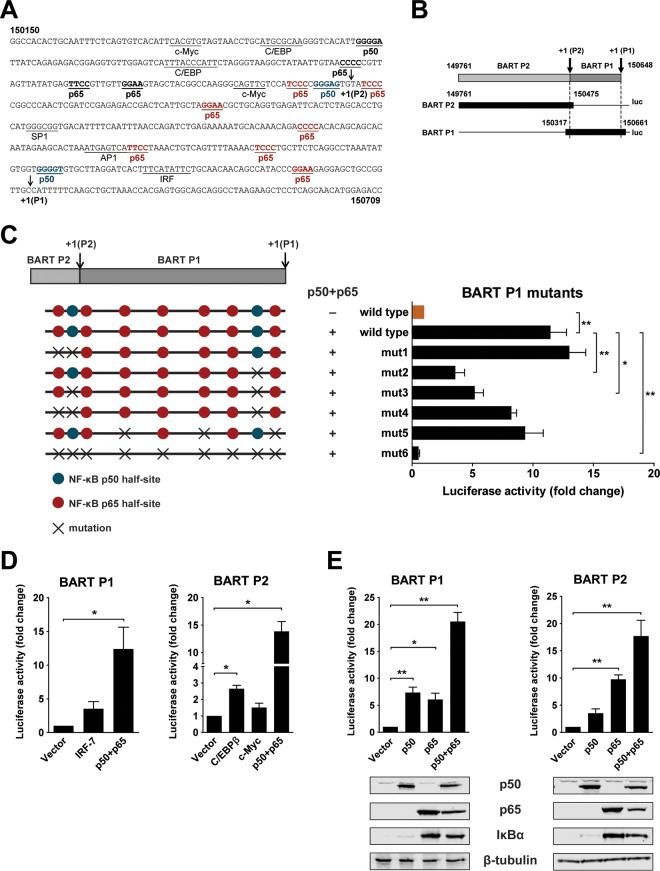
NF-κB activates EBV BART promoters. (A) Sequence of the BART promoter region showing the locations of previously identified transcription factor binding sites (underlined), putative NF-κB binding sites (bold and underlined, with p65 and p50 sites within the region included in the BART P1 reporter shown in red and blue, respectively), and P1 and P2 RNA initiation sites (arrows). The nucleotide coordinates, 150150 to 150709, are based on the EBV B95-8 genome. (B) Illustration of P1 and P2 reporter constructs (solid black). (C) Location of mutations introduced into the P1 reporter at putative NF-κB p50 (blue) and p65 (red) half-sites. HEK 293T cells were cotransfected with p50 and p65 expression vectors together with the BART P1 promoter reporter with and without mutations. Relative luciferase activity is expressed as the fold change compared to the level of wild-type reporter activity, which was set to 1. (D) HEK 293T cells were cotransfected with empty vector or expression vectors for either p50 and p65, IRF-7, C/EBPβ, or c-Myc, together with either the BART P1 or P2 promoter reporter, as indicated. Relative reporter activity was estimated and expressed as the fold change compared to the vector control. (E) HEK 293T cells were transfected with either p50, p65, or p50 and p65 expression vectors together or with a control empty vector, together with the BART P1 or P2 promoter reporter. Relative reporter activity was estimated and expressed as the fold change over that of the vector control. The expression of p50, p65, IκBα, and β-tubulin was confirmed by immunoblotting using specific antibodies. Relative luciferase activities are expressed as the fold change in luciferase activity, calculated by normalizing firefly/Renilla ratios to those of the vector control. Experiments were performed in triplicate, and the results presented are the averages and standard errors of the means from three independent experiments. *P* values were calculated using a two-tailed Student's *t* test (*, *P* < 0.05; **, *P* < 0.01).

We previously identified two promoters which drive expression of BARTs and characterized host factors which regulate the activity of these promoters. Further examination of BART promoter sequences revealed that there are several potential binding sites for NF-κB (Fig. 1A). To test if these motifs mediate activation of BART promoters via NF-κB, we constructed reporters which contained either the BART P1 or BART P2 region and the corresponding NF-κB sites (Fig. 1B). Mutations introduced into different combinations of binding sites caused various degrees of attenuation of activation of the BART promoters by NF-κB; activation of BART promoter activity was almost completely abolished when all sites were mutated (Fig. 1C). The effect of NF-κB p50 and p65 subunits on the BART P1 and P2 promoters was examined in HEK 293T cells in a transfection experiment. Overexpression of p50 and p65 resulted in an excess of dimers relative to the level of cellular IκB inhibitors, resulting in more free and active dimers (33). Results obtained here confirm our previous findings that C/EBPβ and c-Myc act as positive regulators for BART expression (25). NF-κB p50 and p65 were cotransfected individually or together into HEK 293T cells with either a BART P1 or P2 reporter plasmid to further characterize their roles in BART promoter activation. Both p50 and p65 activated the P1 and P2 promoters. Notably, overexpression of p50 and p65 together strongly activated the BART reporters to a greater extent than expression of either C/EBPβ or c-Myc (Fig. 1D and E). These results indicate that BART promoters contain NF-κB binding sites and that NF-κB signaling is a dominant regulator for BART promoter activity in cells.

### NF-κB p50 binds the BART promoters *in vitro* and *in vivo*.

**FIG 2:**
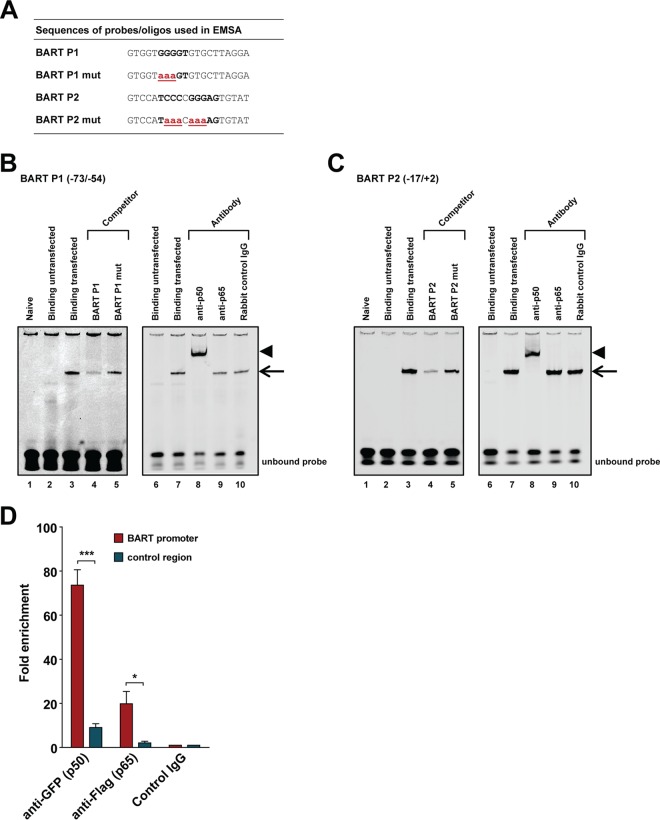
NF-κB binds to NF-κB elements in the BART promoters. (A) Nucleotide sequences of the double-stranded probes and oligonucleotides used in the competition experiments shown below. The NF-κB binding sites in the BART P1 and P2 promoters, consisting of NF-κB half-site sequence(s), are shown in bold uppercase letters, and the mutated nucleotides are in red bold lowercase letters and underlined. (B and C) IRDye 700-labeled double-stranded synthetic oligonucleotides corresponding to the BART P1 region from position −73 to −54 or the BART P2 region from position −17 to +2, as indicated, of the B95-8 sequence were incubated with nuclear extract from HEK 293T cells transfected with plasmids expressing EGFP-tagged p50 and Flag-tagged p65 and subjected to EMSA. The black arrow indicates the NF-κB/probe complex on the gel. Lanes 2 and 6 show the binding pattern of nuclear extract from untransfected cells, while lanes 3 and 7 show the binding pattern of nuclear extract from p50/p65 transfected cells. Lanes 4 and 5 show the binding of the probe when an excess (300-fold) of unlabeled wild-type or mutant competitor oligonucleotide was added to the binding reaction mixture. Supershift experiments with antibodies were performed as indicated above the gels, with supershifted complexes indicated by an arrowhead in lanes 8 of panels B and C. (D) ChIP assay of NF-κB p50 and p65 with the BART promoter. HEK 293T cells were transfected with a plasmid containing the BART P1 promoter sequence and EGFP-tagged p50 and Flag-tagged p65. Results of real-time PCR analysis and ChIP assays with antibodies specific for GFP or Flag or rabbit control IgG are shown. The results are expressed as fold enrichment, where the value for the rabbit control IgG was set to 1. A genomic region ∼5 kb upstream of the IL-8 promoter was used as a negative control. The averages and standard errors of the means from three independent experiments are shown, with all samples being analyzed in triplicate. *P* values were obtained from a two-tailed Student's *t* test (*, *P* < 0.05; ***, *P* < 0.001).

We further tested if the p50 and p65 NF-κB subunits are able to bind to the BART promoters. An electrophoresis mobility shift assay (EMSA) was performed using IRDye 700-labeled probes covering the BART P1 (BART P1 −73/−54) and BART P2 (BART P2 −17/+2) NF-κB sites and nuclear extract prepared from HEK 293T cells transfected with GFP-tagged p50 and FLAG-tagged p65 expression plasmids (Fig. 2A). Protein-DNA complexes could be detected with nuclear extracts prepared from cells transfected with p50 and p65 (Fig. 2B and C, lanes 3) but not with nuclear extracts from untransfected cells. Unlabeled wild-type (WT) probes showed higher competitive ability than probes containing mutations in the NF-κB site (lanes 4 and 5), suggesting that NF-κB subunit binding is associated with the putative NF-κB motifs. The inability of excess unlabeled probe to completely outcompete labeled probe may be due to high affinity of p50/p65 for the NF-κB sites, but this idea needs to be further evaluated with other NF-κB sites. To further verify the specificity of the shifted protein-DNA complex, supershift assays with antibodies to either p50 or p65 were performed. The complex was supershifted with a p50 antibody (Fig. 2B, lane 8), confirming the presence of p50 in the complex. However, the p65 antibody failed to shift the complex, which could be due to the putative NF-κB site consisting of only a p50 half-site, possibly preventing p65 antibody binding to the complex (Fig. 2B and C, lanes 9). It is also possible that the anti-p65 antibody chosen is not suitable for use in the supershift assay as the BART P2 complex is also supershifted by anti-p50 but not by anti-p65 (Fig. 2C, lanes 8 and 9). Rabbit control IgG antibody did not result in any supershifted complexes, supporting the specificity of the supershift reactions (Fig. 2B and C, lanes 10). The absence of a supershift with the p65 antibody in the EMSA with both BART P1 and P2 probes may suggest that p50 is a key subunit for NF-κB binding to BART promoters.

To further verify the role of NF-κB signaling in regulation of EBV BART promoters, a chromatin immunoprecipitation (ChIP) assay was performed to explore the ability of p50 and p65 to bind the putative NF-κB binding sites in the BART promoters *in vivo*. GFP-tagged p50 and Flag-tagged p65 were cotransfected with the P1 reporter into HEK 293T cells, and cell lysates were precipitated with either anti-GFP, anti-Flag, or control IgG. The p50 (via anti-GFP)-enriched BART promoter DNA in the precipitates appeared to be almost 80-fold more abundant than that precipitated by rabbit control IgG, while for p65 (via anti-Flag), a moderate enrichment of approximately 20-fold over the control antibody was observed (Fig. 2D). The lower enrichment for p65 than that for p50 in the ChIP experiment supports the suggestion that p50 dictates the interaction with BART promoter NF-κB motifs. These results suggest that NF-κB p50 and, to a lesser extent, p65 are able to bind to the BART promoters *in vitro* and *in vivo*.

### LMP1 activates BART promoters through NF-κB signaling.

**FIG 3:**
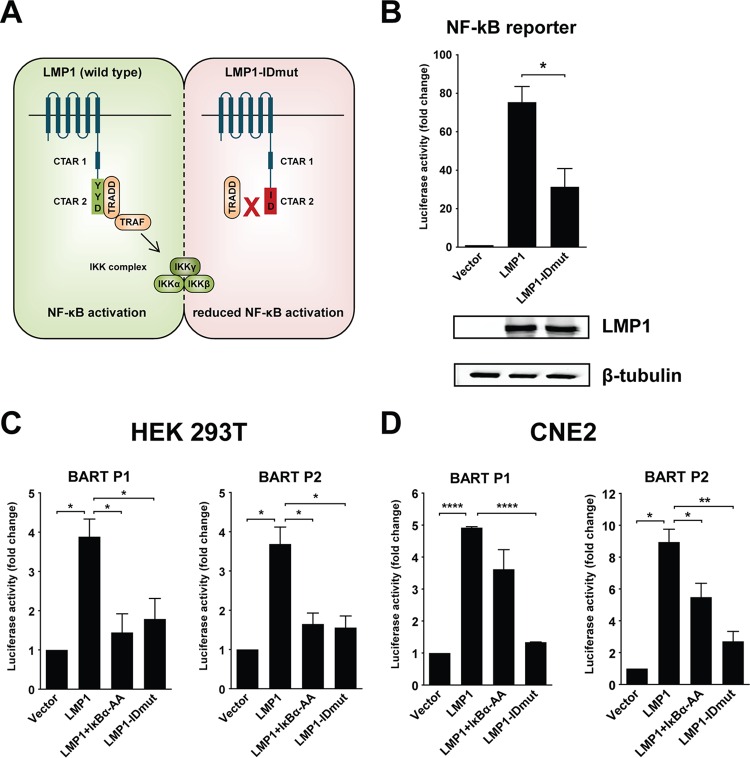
LMP1 expression activates BART P1 and P2 reporters through NF-κB signaling. (A) Illustration of wild-type LMP1 and the mutated LMP1 showing amino acid changes in CTAR2 from YYD to ID, resulting in reduced NF-κB activation by LMP1, as described in a previous study (38). (B) HEK 293T cells were transfected with an NF-κB-responsive reporter, together with an empty vector or an LMP1 (wild-type or mutant) expression vector, and reporter activities were determined 24 h after transfection. The expression of LMP1 and β-tubulin was detected by immunoblotting using specific antibodies. (C and D) HEK 293T and CNE2 cells were transfected with an empty vector or an LMP1 (wild-type or mutant) expression vector, with or without an IκBα-AA expression vector, together with the BART P2 or P1 promoter reporter. Luciferase activities are expressed as the fold change in luciferase activity, calculated by normalizing firefly/Renilla ratios to those of the vector control. Experiments were performed in triplicate, and the results are the averages and standard errors of the means from at least three independent experiments. *P* values were calculated by two-tailed Student's *t* test (*, *P* < 0.05; **, *P* < 0.01; ****, *P* < 0.0001).

Since LMP1 is a known activator of NF-κB signaling, we tested if LMP1 can activate the BART promoters through this pathway. For this purpose we first made an LMP1 mutant (LMP1-IDmut) with impaired NF-κB activation ability by mutating the YYD amino acid sequence in C-terminal activating region 2 (CTAR2) of LMP1 to ID (Fig. 3A) (38). The impaired ability of LMP1-IDmut to induce NF-κB activation was confirmed using an NF-κB reporter (Fig. 3B). Immunoblot analysis using LMP1-specific antibodies confirmed expression of the LMP1 mutant in transfected cells (Fig. 3B, bottom panel). Reporter assays with the BART promoters showed that LMP1 could activate both BART P2 and P1 approximately 4-fold more strongly than the control in HEK 293T cells (Fig. 3C). Cotransfection of LMP1 with IκBα-AA, an IκBα mutant which cannot be phosphorylated, abolished activation of BART promoters by LMP1, confirming that the positive effect on BART promoters is mediated by NF-κB signaling. The role of NF-κB signaling in LMP1 activation of BART promoters was further confirmed using LMP1-IDmut, which showed attenuated ability to activate BART promoters (Fig. 3C). Activation of BART promoters by LMP1 through NF-κB signaling also occurred in NPC CNE2 cells, with upregulation also being diminished with LMP1-IDmut or coexpression of LMP1 with IκBα-AA (Fig. 3D). Taken together, these results show that LMP1 can activate the BART promoters, largely through NF-κB signaling.

**FIG 4:**
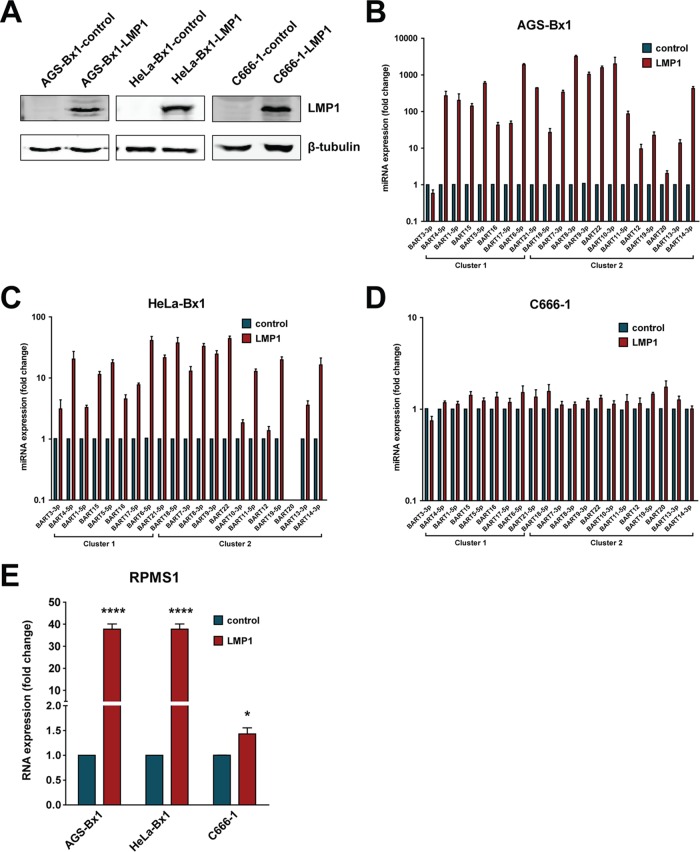
Stable expression of LMP1 in EBV-infected epithelial cell lines upregulates BART expression. (A) LMP1 protein expression from the AGS-Bx1-LMP1, HeLa-Bx1-LMP1, and C666-1-LMP1 stable cell lines was examined by immunoblotting to demonstrate the overexpression of LMP1. (B to D) Levels of BART miRNA expression in control and LMP1-expressing stable cell lines were determined by real-time PCR. BART miRNA expression was normalized to that of a host miRNA, miR-Hsa-16, and is presented as fold change, with the expression levels of the control cell line being set to 1. (E) Levels of BART RPMS1 RNA expression in control and LMP1-expressing stable cell lines were determined by real-time PCR using primers described in Table 1. RPMS1 RNA expression was normalized to that of GAPDH and is presented as fold change, where the expression of the negative-control cell line was set to 1. Real-time PCR experiments were performed in triplicate, and the results are the averages and standard errors of the means from at least three independent experiments. *P* values were calculated using a two-tailed Student's *t* test (*, *P* < 0.05; ****, *P* < 0.0001).

To further confirm regulation of BART expression by LMP1, we stably expressed LMP1 in the EBV-infected epithelial cell lines AGS-Bx1, HeLa-Bx1, and NPC C666-1 and then compared the expression levels of BART miRNAs and lncRNAs. LMP1 expression was confirmed in these cell lines compared to expression in vector control cell lines (Fig. 4A). Overexpression of LMP1 in AGS-Bx1 and HeLa-Bx1 cells stably expressing LMP1 resulted in significant upregulation of BART lncRNAs. Examination of miRNA expression profiles revealed that most BART miRNAs were upregulated to various degrees, with the only exception being miR-BART3-3p (Fig. 4B and C). Expression of BARTs comprises both BART miRNAs and BART lncRNAs. Examination of BART lncRNAs with a pair of primers specific for the RPMS1 region found that BART RNA was upregulated almost 40-fold in both the AGS-Bx1-LMP1 and HeLa-Bx1-LMP1 cell lines (Fig. 4E). Notably, additional expression of LMP1 in C666-1 cells did not result in a similar level of upregulation of BART expression (Fig. 4D and E). It is possible that C666-1 cells contain constitutively active NF-κB and that any additional effect resulting from LMP1 expression, which is mediated by the same signaling pathway, may be masked. These results further support the positive effect of LMP1 on BART expression, presumably through NF-κB signaling, in EBV-infected epithelial cells.

### EBV-harboring C666-1 NPC cells contain constitutively activated NF-κB and express higher levels of BARTs than other EBV-infected epithelial cell lines.

**FIG 5:**
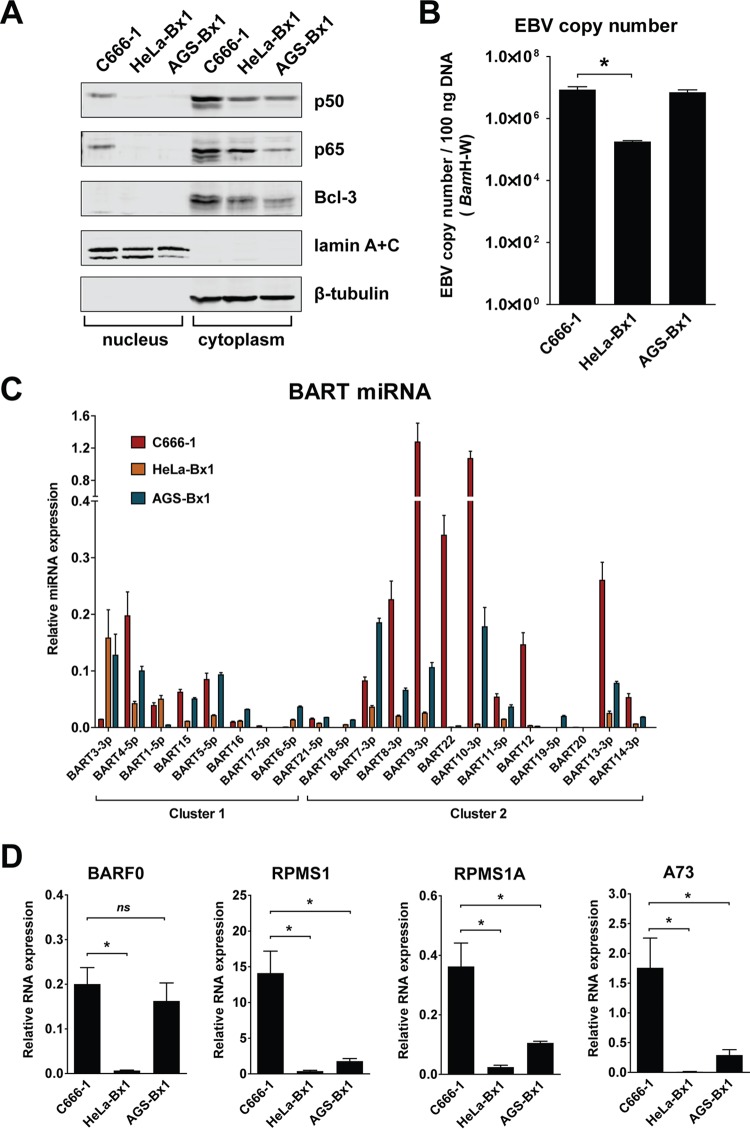
NF-κB activation and BART RNA and miRNA expression in EBV-infected epithelial cell lines. (A) Immunoblot analysis of nuclear and cytoplasmic fractions from the cell lines C666-1, HeLa-Bx1, and AGS-Bx1, using specific antibodies. Antibodies against lamin A+C were used to verify preparation of nuclear and cytoplasmic fractions. (B) EBV copy numbers in the cell lines C666-1, HeLa-Bx1, and AGS-Bx1 were determined by amplifying a fragment of the BamH-W region using qPCR. (C) Relative levels of BART miRNA in the EBV-infected epithelial cell lines C666-1, HeLa-Bx1, and AGS-Bx1 were determined by real-time PCR. BART miRNA expression was normalized to that of miR-Hsa-16. (D) Relative levels of the alternatively spliced BART RNAs BARF0, RPMS1, RPMS1A, and A73 in the EBV-infected epithelial cell lines C666-1, HeLa-Bx1, and AGS-Bx1 were determined by real-time PCR. BART RNA expression was normalized to that of GAPDH. Real-time PCR experiments were performed in triplicate, and the results are the averages and standard errors of the means from three independent RNA extractions. *P* values were calculated using a two-tailed Student's *t* test (*, *P* < 0.05; ns, not significant).

Expression of EBV BART miRNAs is reported to be deregulated in EBV-associated neoplasia (24). We previously found that EBV-harboring C666-1 NPC cells consistently express higher levels of miRNAs than EBV-infected NP-460-Tert-EBV cells (37). This suggests that specific cellular conditions may facilitate the high-level expression of BART noncoding RNAs and miRNAs in NPC cells. To examine this idea, we further compared BART expression levels in EBV-harboring C666-1 NPC, AGS-Bx1, and HeLa-Bx1 cells. NF-κB signaling is constitutively activated in NPC cells (28, 29). We found that C666-1 cells, but not AGS-Bx1 or HeLa-Bx1 cells, contained nuclear p50 and p65 (Fig. 5A). While HeLa-Bx1 cells contained lower copy numbers of the EBV genome, C666-1 and AGS-Bx1 cells harbored similar EBV copy numbers (Fig. 5B). We found that C666-1 cells consistently expressed higher levels of BART lncRNAs and miRNAs than either AGS-Bx1 or HeLa-Bx1 cells (Fig. 5C and D). Interestingly, higher levels of Bcl-3, which is upregulated by NF-κB signaling, were also observed in C666-1 cells (Fig. 5A). We have shown above that NF-κB signaling is important for BART promoter activity. While it is suggested that Bcl-3 may interact with NF-κB, it remains to be evaluated if it is associated with the elevated levels of BART observed in NPC cells. These data further strengthen the argument that NF-κB signaling contributes to BART expression in NPC cells.

### Inhibition of NF-κB signaling in C666-1 cells downregulates BART expression.

**FIG 6:**
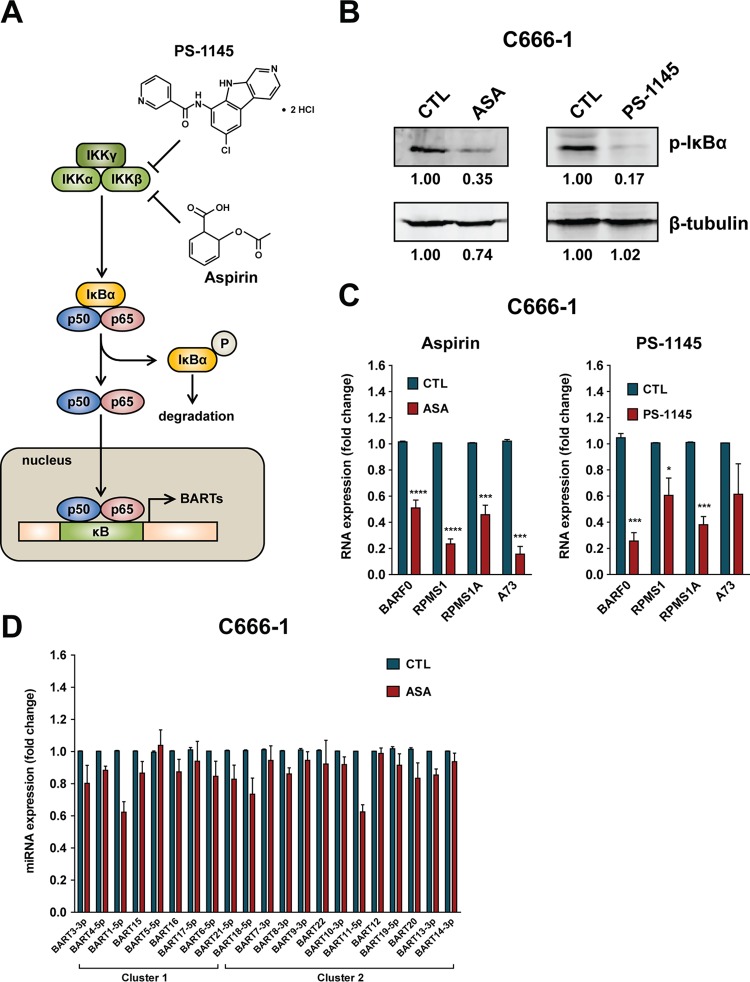
Inhibition of NF-κB signaling using acetylsalicylic acid (ASA) and the IκB kinase inhibitor PS-1145 downregulates BART expression in C666-1 cells. (A) Schematic showing the process of inhibition of NF-κB signaling by ASA and PS-1145. (B) Immunoblot analysis of levels of phosphorylated IκBα protein from C666-1 cells treated with either an ethanol (for ASA) or dimethyl sulfoxide (for PS-1145) control (CTL), 4 mM ASA, or 0.2 mM PS-1145 for 48 h. The protein levels were quantified using Image Studio Lite software (Li-Cor) and normalized against the solvent control treatment. (C) Levels of BART lncRNAs from spliced forms of BARF0, RPMS1, RPMS1A, and A73 RNA in C666-1 cells after treatment with either ASA, PS-1145, or solvent control for 48 h were determined by real-time PCR. BART RNA expression was normalized to that of GAPDH and is presented as fold change, where the value for the solvent control was set to 1. (D) Expression of BART miRNA in C666-1 cells after treatment with either ASA or solvent for 48 h was determined by real-time PCR. BART miRNA expression was normalized to that of miR-Hsa-16 and is presented as fold change, with the value for the ethanol control set to 1. Real-time PCR experiments were performed in triplicate, and the results are the averages and standard errors of the means from three independent experiments. *P* values were calculated from a two-tailed Student's *t* test comparison of results from NF-κB inhibitor treatment to those with control treatment (*, *P* < 0.05; ***, *P* < 0.001; ****, *P* < 0.0001).

To further confirm the idea that NF-κB activity is critical for BART expression, we tested if inhibition of NF-κB activity would affect levels of BART in NPC cells. Acetylsalicylic acid (ASA; aspirin) is an anti-inflammatory drug which inhibits cyclooxygenase and IκBα (39–41), which is associated with NF-κB signaling (Fig. 6A). A previous study also reported that the IκB kinase (IKK) inhibitor PS-1145 specifically blocks TNF-α-induced NF-κB activation through inhibition of IκBα phosphorylation and degradation (42). When C666-1 cells were treated with either ASA or PS-1145, immunoblot analysis showed a decrease in phosphorylated IκBα, indicating reduced NF-κB activation (Fig. 6B). Consistent with the decrease in NF-κB activity, ASA and PS-1145 treatment significantly downregulated the expression of all tested spliced forms of BART lncRNAs, including BARF0, RPMS1, RPMS1A, and A73 (Fig. 6C). Furthermore, examination of BART miRNA expression revealed lower levels of most BART miRNAs in ASA-treated C666-1 cells (Fig. 6D). These results demonstrate that interference with NF-κB signaling causes downregulation of EBV BART expression, further confirming that BART expression is regulated by NF-κB signaling.

### BART miRNA targets the LMP1 3′ UTR to downregulate LMP1 protein expression.

**FIG 7:**
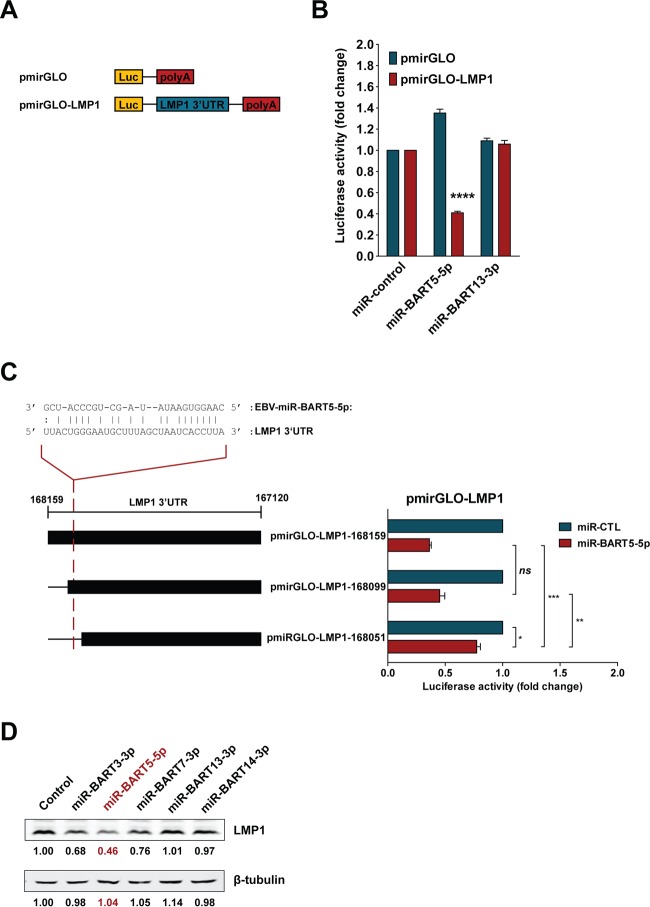
EBV-encoded miR-BART5-5p modulates LMP1 expression. (A) The pmirGLO reporter plasmid constructs used to assess the effect of miR-BART5-5p on the LMP1 3′ UTR. (B) HEK 293T cells were transfected with pmirGLO-LMP1 or empty pmirGLO reporter, together with 50 nM total synthetic miRNA (miR-control), miR-BART5-5p, or miR-BART13-3p duplexes. Reporter activity was determined 24 h after transfection. Firefly/Renilla ratios were normalized against the same reporter transfected with the negative-control miRNA, and results are expressed as fold change in luciferase activity. (C) Sequence alignment of miR-BART5-5p and the predicted binding site on the LMP1 3′ UTR (35). HEK 293T cells were cotransfected with a pmirGLO-LMP1 or pmirGLO-truncated LMP1 3′ UTR reporter and either 50 nM miR-CTL or miR-BART5-5p. Firefly/Renilla ratios were normalized to levels of the same reporter transfected with miR-CTL and expressed as fold change in luciferase activity. The results are the averages and standard errors of the means from three independent experiments. *P* values are from a two-tailed Student's *t* test comparison of results with the BART miRNA to those with the control miRNA (*, *P* < 0.05; **, *P* < 0.01; ***, *P* < 0.001; ns, not significant). (D) HEK 293T cells stably expressing LMP1, including its 3′ UTR, were transfected with 50 nM synthetic miRNA (control), miR-BART3-3p, miR-BART5-5p, miR-BART7-3p, miR-BART13-3p, or miR-BART14-3p duplexes. Expression of LMP1 was detected by immunoblotting using specific antibodies, and the intensities of bands were determined using Image Studio Lite software. Levels of β-tubulin were determined as a loading control. The intensities of the bands were normalized to the level of the negative-control miRNA and are expressed as fold change in protein expression.

Expression of LMP1 in NPC cells is regulated by host mechanisms (30, 43). Because cells expressing LMP1 may be targeted by immune surveillance, it is believed that expression of LMP1 is tightly regulated. Previous studies showed that EBV BART miRNA could downregulate LMP1 expression (34, 35). It seems possible that LMP1 and BARTs form an autoregulatory loop in maintaining EBV latency in NPC. To confirm whether BART miRNA may serve as a negative regulator to modulate LMP1 protein expression, we analyzed the targeting and downregulation of LMP1 by miR-BART5-5p, which has an imperfect sequence match to the LMP1 3′ UTR (Fig. 7A). Reporters containing the entire LMP1 3′ UTR followed by the poly(A) signal or controls were used in coexpression experiments with EBV-miR-BART5-5p (Fig. 7B). EBV-miR-BART5-5p significantly downregulated luciferase activity for the reporter containing the LMP1 3′ UTR, unlike miR-BART13-3p, which has no sequence matches to the LMP1 3′ UTR region and showed no downregulation effect (Fig. 7B). Interestingly, a truncated form of the LMP1 3′ UTR which lacks the reported EBV-miR-BART5-5p targeting site was also downregulated by EBV-miR-BART5-5p, albeit less strongly, suggesting that there may be multiple BART miRNA-targeting sites within the LMP1 3′ UTR (Fig. 7C). Similarly, immunoblot analysis of HEK 293T cells stably expressing LMP1 containing the 3′ UTR showed downregulation of LMP1 protein expression by miR-BART5-5p and, to a lesser extent, by miR-BART3-3p and miR-BART7-3p but not by miR-BART13-3p or miR-BART14-3p (Fig. 7D). These results confirm the ability of BART miRNA to downregulate LMP1 and thereby form a negative feedback loop to modulate expression of LMP1 in EBV-infected NPC cells.

## DISCUSSION

Epstein-Barr virus ubiquitously expresses a family of BamHI-A rightward transcripts (BARTs), which are composed of alternatively spliced forms of lncRNAs and two clusters of miRNAs derived from the BamHI-A region of the viral genome, in all forms of latency (12–14). Functions associated with BART miRNAs and lncRNAs are being revealed, and expression of BART miRNA is known to be deregulated in EBV-associated tumors (20, 22, 23, 24, 44). It is therefore necessary to understand how the expression of BARTs is regulated in EBV-infected cells, in particular, in the setting of EBV-associated tumors. We previously determined that BARTs are expressed from two promoters and can be positively regulated by C/EBP and c-Myc (25). This study further demonstrated that NF-κB signaling plays a crucial role in activation of expression of BARTs. Activation of NF-κB signaling by expression of LMP1 or interference with NF-κB signaling using chemicals that inhibit phosphorylation of IκBα resulted in positive and negative modulation of BART expression, respectively. We propose that EBV may utilize an autoregulatory loop to maintain expression of BARTs through constitutively activated NF-κB signaling while keeping LMP1-mediated NF-κB signaling in check through BART miRNA modulation in infected cells.

The most distinctive feature of EBV latency in NPC is the expression of high levels of BARTs, which include BART lncRNAs and miRNAs, but few other latent products. It is reasonable to postulate that the host mechanism which regulates the expression of BARTs might be associated with the oncogenic state in NPC. Based on our previous work identifying two promoters for the expression of BARTs (25), this study set out to characterize the aberrant signals which contribute to the expression of BARTs in NPC. BARTs are expressed from two TATA-less promoters (P1 and P2). The BART P1 promoter contains a C/EBP motif, and C/EBP was found to exert a positive effect on BART expression (25). Since C/EBP is commonly expressed in epithelial cells, it seems likely that other signaling pathways associated with the malignant status of cancer cells may contribute to the deregulation of expression of BARTs in NPC. In this study, we identified several potential NF-κB sites between positions −3 (P2) and −69 (P1) in the BART promoters and demonstrated binding of NF-κB p50 to the BART promoters. While the BART promoters contain only half of the NF-κB p50 consensus sequence, these sites seem sufficient for NF-κB p50 binding and mediation of NF-κB signaling to activate promoter activity. The fact that both NF-κB sites in BART P1 and P2 are p50 consensus half-sites may explain why the ChIP assay is more efficient with p50 than with p65 and why only a supershift with the p50 antibody was observed in EMSAs. Nevertheless, coexpression of p50 and p65 resulted in significant activation of BART promoter activity, suggesting that the p50/p65 heterodimer is important for BART promoter regulation. While C/EBP and c-Myc were found to positively regulate BART expression, we showed that NF-κB signaling has a more potent effect and may be the dominant regulator for both BART promoters. It is still unclear if P1 and P2 may be responsible for expression of different species of BART lncRNAs or miRNAs.

**FIG 8:**
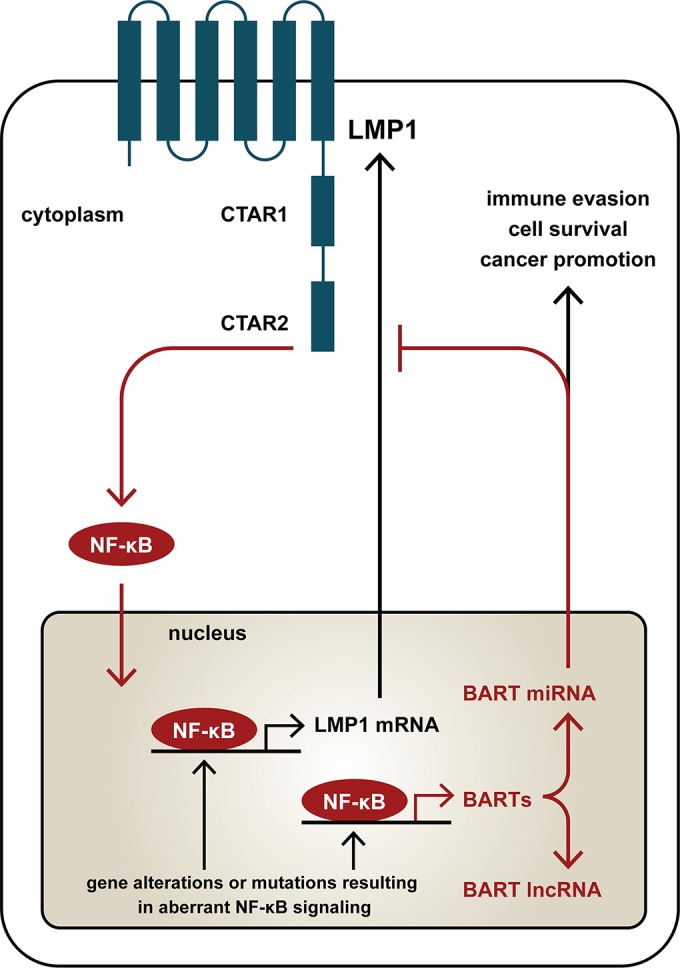
Working model showing a regulatory loop between NF-κB, LMP1, and BART miRNA in NPC cells. Constitutively active NF-κB, together with LMP1 produced through NF-κB signaling, upregulates LMP1 and BART miRNA and lncRNA expression in EBV-infected NPC cells. BART miRNA can downregulate LMP1 expression by targeting its 3′ UTR and thereby forms a negative feedback loop to modulate the level of LMP1 in cancer cells. The aberrant NF-κB activity in EBV-infected NPC cells ensures high-level expression of BART miRNAs and lncRNAs, which may contribute to immune evasion, cell survival, and cancer promotion.

Constitutively activated NF-κB is implicated in the initiation and progression of human cancers through its role in regulating cell proliferation, apoptosis, cell transformation, and immunosuppression (45, 46). In nasopharyngeal cells, infection with EBV can activate the NF-κB pathway, which likely contributes to tumor progression (47, 48). Interestingly, gene alterations or mutations in components involved in NF-κB pathways have been observed in NPC tumor lines and primary tumor tissues (29). High levels of NF-κB have been demonstrated to inhibit gammaherpesvirus lytic replication in both lymphoid and epithelial cells, while virus reactivation in NPC could be achieved by inhibiting NF-κB (49, 50). It seems possible that predisposing genetic conditions may permit constitutively activated NF-κB signals in precancerous cells that facilitate EBV latency and that expression of LMP1 following virus infection may further enhance aberrant NF-κB signals, leading to NPC malignancy. Besides being able to activate NF-κB, LMP1 expression was also found to be upregulated by NF-κB, resulting in a positive regulatory loop between LMP1 expression and NF-κB signaling (33). We previously determined that aberrant STAT activity also contributes to the expression of LMP1 and Qp-EBNA1 in NPC cells (30, 43). These findings point to a mechanism in which the combined effects of genetic alteration and EBV infection may jointly trigger the onset or progression of NPC. While shedding of lytic replicating EBV is regularly detected in saliva from positive carriers, EBV remains in a stringent latent state in NPC cells. It may be speculated that predisposing conditions, such as constitutively activated STAT3 and NF-κB, in some “precancerous” NP cells may facilitate EBV latency through expression of EBV EBNA1, LMP1, and high levels of BART miRNAs and lncRNAs. EBV-encoded BART miRNAs have been shown to target LMP1 and may prevent accumulation of overly high levels of LMP1 that would otherwise lead to apoptosis or induce an immune response (34, 35). Here, we further confirmed the linkage between LMP1 and NF-κB by showing that expression of BARTs is upregulated by NF-κB, that LMP1 positively regulates BART miRNA expression through NF-κB signaling, and that expression of BART miRNAs can downregulate LMP1 expression to provide negative feedback. Maintaining high levels of BART miRNAs and lncRNAs in cancer cells could have complex functions in cancer promotion, cell survival, and immune evasion (Fig. 8).

In addition to the upregulation of BART miRNAs, we also found that NF-κB signaling regulated the alternatively spliced BART lncRNAs, which was to be expected since BART miRNA levels have been linked to the transcription of BART RNAs (17). The exact function of BART lncRNAs remains to be elucidated since no study has been able to detect endogenous protein expression from the BART open reading frames in EBV-infected cells (19, 51–53). A recent study reported that expression of BART lncRANs altered cellular gene expression, suggesting a potential function as yet undefined (20). This study provides evidence that reemphasizes the critical role of NF-κB in modulating EBV latency in NPC. The cyclooxygenase 2 inhibitor ASA has been reported to induce EBV lytic replication by inhibiting NF-κB activity, resulting in cytotoxicity which could be amplified by ganciclovir (49). The IκB kinase inhibitor PS-1145 was shown to block TNF-α-induced NF-κB activation in multiple myeloma cells through inhibition of IκBα phosphorylation and degradation (42). By treating C666-1 cells with ASA or PS-1145, we were able to inhibit NF-κB activity, resulting in downregulation of BART RNA expression. The fact that inhibition of NF-κB signaling both downregulates BART expression and induces lytic replication makes the NF-κB pathway a potential target for future studies exploring novel options for the treatment of EBV-associated carcinomas.
